# Psychometric performance of the Chichewa versions of the EQ-5D-Y-3L and EQ-5D-Y-5L among healthy and sick children and adolescents in Malawi

**DOI:** 10.1186/s41687-023-00560-4

**Published:** 2023-03-09

**Authors:** Lucky G. Ngwira, Hendramoorthy Maheswaran, Janine Verstraete, Stavros Petrou, Louis Niessen, Sarah C. Smith

**Affiliations:** 1 Malawi-Liverpool-Wellcome Trust Clinical Research Programme, Chipatala Avenue, P.O. Box 30096, Chichiri, Blantyre 3, Malawi; 2 Department of Clinical Sciences, Liverpool School of Tropical Medicine, Liverpool, UK; 3 Imperial College London, London, UK; 4 University of Cape Town, Cape Town, South Africa; 5 University of Oxford, Oxford, UK; 6 John Hopkins School of Public Health, Baltimore, MD USA; 7 London School of Hygiene and Tropical Medicine, London, UK

**Keywords:** EQ-5D-Y-5L, EQ-5D-Y, Childhood, Health-related quality of life, HRQoL, Translation, Adaptation, Ranking exercise, Sub-Saharan Africa

## Abstract

**Objectives:**

The EuroQol Group has developed an extended version of the EQ-5D-Y-3L with five response levels for each of its five dimensions (EQ-5D-Y-5L). The psychometric performance has been reported in several studies for the EQ-5D-Y-3L but not for the EQ-5D-Y-5L. This study aimed to psychometrically evaluate the EQ-5D-Y-3L and EQ-5D-Y-5L Chichewa (Malawi) versions.

**Methods:**

The EQ-5D-Y-3L, EQ-5D-Y-5L and PedsQL™ 4.0 Chichewa versions were administered to children and adolescents aged 8–17 years in Blantyre, Malawi. Both of the EQ-5D-Y versions were evaluated for missing data, floor/ceiling effects, and validity (convergent, discriminant, known-group and empirical).

**Results:**

A total of 289 participants (95 healthy, and 194 chronic and acute) self-completed the questionnaires. There was little problem with missing data (< 5%) except in children aged 8–12 years particularly for the EQ-5D-Y-5L. Ceiling effects was generally reduced in moving from the EQ-5D-Y-3L to the EQ-5D-Y-5L. For both EQ-5D-Y-3L and EQ-5D-Y-5L, convergent validity tested with PedsQL™ 4.0 was found to be satisfactory (correlation ≥ 0.4) at scale level but mixed at dimension /sub-scale level. There was evidence of discriminant validity (*p* > 0.05) with respect to gender and age, but not for school grade (*p* < 0.05). For empirical validity, the EQ-5D-Y-5L was 31–91% less efficient than the EQ-5D-Y-3L at detecting differences in health status using external measures.

**Conclusions:**

Both versions of the EQ-5D-Y-3L and EQ-5D-Y-5L had issues with missing data in younger children. Convergent validity, discriminant validity with respect to gender and age, and known-group validity of either measures were also met for use among children and adolescents in this population, although with some limitations (discriminant validity by grade and empirical validity). The EQ-5D-Y-3L seems particularly suited for use in younger children (8–12 years) and the EQ-5D-Y-5L in adolescents (13–17 years). However, further psychometric testing is required for test re-test reliability and responsiveness that could not be carried out in this study due to COVID-19 restrictions.

**Supplementary Information:**

The online version contains supplementary material available at 10.1186/s41687-023-00560-4.

## Introduction

The adult EQ-5D-3L, is one of the most widely used preference-based health-related quality of life (HRQoL) measures for health economic evaluations [1]. Despite this prominence, the EQ-5D-3L has been criticized for its simplicity and insensitivity to small changes in health status, leading to the development of the five response level, the EQ-5D-5L [2]. Evidence suggests that the EQ-5D-5L performs better, is less affected by ceiling effects and improves known-group validity compared to the EQ-5D-3L [3–5].

The youth friendly three response level, EQ-5D-Y-3L, and the experimental five response level EQ-5D-Y-5L have emerged from the adult EQ-5D versions [6, 7]. The EQ-5D-Y-5L was developed on the same premise as the adult EQ-5D-5L version to increase sensitivity and reduce ceiling effects [2]. Psychometric performance of the EQ-5D-Y-3L has been reported in studies involving children with different health conditions [8–10]. To a large extent, it has demonstrated good reliability, with acceptable levels of convergent, discriminant and known-group validity [11–13], but has reported problems with missing values [14]. The performance of the newly developed EQ-5D-Y-5L has only been reported in a small number of studies [15–22]. The EQ-5D-Y-5L has demonstrated feasibility and minimal ceiling effects in these studies, but it has not performed differently on other psychometric properties compared to the EQ-5D-Y-3L [15, 17, 23].

Neither the EQ-5D-Y-3L nor the EQ-5D-Y-5L have been psychometrically evaluated in Malawi where economic evaluation in health programs is becoming increasingly important [24]. This study set out to psychometrically evaluate the Chichewa (Malawi’s national language) versions of the EQ-5D-Y-3L and EQ-5D-Y-5L among children and adolescents.

## Methods

### Participants, recruitment and procedure

The study recruited participants from a convenience sample of healthy and sick children (8–12 years) and adolescents (13–17 years) in urban Blantyre, Malawi. Children and adolescents attending schools and seeking any health care services through out-patient department at the Queen Elizabeth Central Hospital made up healthy and sick participants, respectively. Written assent and consent was obtained from children and their parents/guardians. For sick participants, the invitation came at the end of clinical care. For healthy participants, invitations were made through the school via a teacher. Participants took the study information leaflets and consent forms home for receipt of consent by their respective parents/guardians and these were brought back to the school the following day. For both sets of participants, once consent was obtained, the questionnaires were distributed by the research team at the end of clinical care or interviews were arranged on a school day. Once the participants completed the questionnaires (in clinic or classroom settings, respectively), the forms were handed over and collected by the study staff. Only children who were literate (as evident from the written consenting process) and therefore able to self-complete the questionnaires were included, but the critically ill were excluded from recruitment. As previous research had revealed a tendency for respondents to avoid the middle responses when completing the adult EQ-5D-5L questionnaire if the EQ-5D-3L is administered first [3], the EQ-5D-Y-5L was administered before the EQ-5D-Y-3L. This was followed by the self-report Pediatric Quality of Life (PedsQL)™ 4.0 Generic Core Scales for children (8–12 years) or teens (13–17 years). Ethical approval for this study was granted by Ethics Committees at the Malawi College of Medicine (now KUHeS) (P.10/18/2509) and Liverpool School of Tropical Medicine (19-045). A sample size of 200 participants was calculated to provide 80% power, at the two-sided significance level of 0.05, to address the minimum psychometric criteria for convergent and discriminant validity.

### The instruments

#### The EQ-5D-Y-3L

The EQ-5D-Y-3L consists of five dimensions: ‘mobility’, ‘looking after myself’, ‘doing usual activities’, ‘having pain or discomfort’, and ‘feeling worried, sad or unhappy’. Responses in each dimension are separated into three ordinal levels: (1) no problems, (2) some problems /a bit, and (3) a lot of problems/very. Self-rated health status was also assessed with the measure’s visual analogue scale (EQ VAS), a vertical scale with scores ranging between 0 (representing worst imaginable health) and 100 (representing best imaginable health). The EQ-5D-Y has a same day recall period [6].

#### The EQ-5D-Y-5L

The EQ-5D-Y-5L consists of the same five dimensions as the EQ-5D-Y-3L but with five responses each: (1) no problems/not, (2) a little bit of a problem, (3) some problems /quite, (4) a lot of problems/really, and (5) extreme problems/extremely/cannot.

The cross-cultural adaptation of both the EQ-5D-Y-3L and EQ-5D-Y-5L into Chichewa has been reported elsewhere [25]. Briefly, this included forward and backward translation, and cognitive debriefing among children and adolescents aged 8–15 years. Sociodemographic and medical data were also recorded for each participant on a separate page.

The EQ-5D-Y-3L and EQ-5D-Y-5L were scored using the sum scores by summing the responses. The sum score is a crude measure with some limitations, but for psychometric evaluation it gives a better indication of the dimension performance [26]. A health state (represented by responses) ‘11111’ (denoting a one for each of the five dimensions) had a level sum score of 5. The sum scores ranged between 5 and 15 (EQ-5D-Y-3L) or 25 (EQ-5D-Y-5L) (lower = better). Secondly, utility scores indexed at 0 and 1 (higher = better) for the EQ-5D-Y-3L and EQ-5D-Y-5L were calculated using value sets for adults as no EQ-5D-Y-5L value sets were available at the time of conducting this study. Few countries have adult value sets for both the EQ-5D-3L and EQ-5D-5L, and none of these are in Africa [27]. Thus, the utility scores were calculated using the adult value sets (for the United States of America (US)) developed by Shaw et al. [28] and Pickard et al. [29], respectively. The 2005 US EQ-5D-3L (n = 4048) value set (range -0.109, 1) used the Measurement and Valuation of Health (MVH) protocol which uses a different approach for states worse than dead, whereas the 2019 US EQ-5D-5L (n = 1134) value set (range -0.573, 1) used a composite time trade-off (cTTO) in estimating utilities.

#### Self-rated general health

A self-rated general health rating was included through the question: How would you rate your health today? Excellent, very good, good, fair, or poor? Although limited, a single question health rating is an efficient measure of health status that can provide a useful comparison [17, 30].

#### The Pediatric Quality of Life ™ version 4.0 Generic Core Scales

The Chichewa versions of the Pediatric Quality of Life™ version 4.0 Generic Core Scales (GCS) child self-report (8–12 years) or the PedsQL™ 4.0 GCS teen self-report (13–18 years) were administered, dependent on the age of the respondent. The translation processes and approvals for these measures were provided by the Mapi Trust [31]. Both the PedsQL™ 4.0 GCS versions (herein referred to as PedsQL™ 4.0 GCS for brevity) have 23 items across four subscales: (1) Physical Functioning (8 items), (2) Emotional Functioning (5 items), (3) Social Functioning (5 items), and (4) School Functioning (5 items). The only difference between the child and teen versions is the use of the terms ‘kids’ or ‘teens’ for some items. Responses for each item are on a 5-point scale coded: (0) never a problem, (1) almost never a problem, (2) sometimes a problem, (3) often a problem, or (4) almost always a problem. Responses are reverse scored and linearly transformed on to a 0–100 scale (0 = 100, 1 = 75, 2 = 50, 3 = 25, 4 = 0). The PedsQL™ 4.0 GCS total scale score is obtained by scoring across all 23 items (higher = better). The Physical Functioning subscale score is obtained by summing the scores for the eight Physical Functioning items, whereas the last three subscales (15 items) are combined to form the Psychosocial Health scale score. The subscale scores are obtained through the summation of scores divided by the number of items answered to give a score ranging from 0–100, thereby accounting for missing responses if present [32, 33].

The cross-cultural adaptation of both the PedsQL™ 4.0 GCS child self-report and PedsQL™ 4.0 GCS teen self-report into Chichewa is being prepared for publication elsewhere. Briefly, this process similarly included forward and backward translation, as well as cognitive debriefing among children and adolescents aged 8–15 years.

### Psychometric analyses

Data analysis were performed using IBM SPSS 26.0.0 for Mac (IBM Corp. Armonk, New York, USA) [34]. The sample was divided into two groups: children and adolescents to reflect the age ranges for the self-report PedsQL™ 4.0 GCS child (8–12 years) and teen (13–18 years) scales. Psychometric analyses were evaluated using these age groups, as well as combined age groups, and by health conditions (acute and chronic).

### General performance and feasibility

The analysis of the EQ-5D-Y-3L and EQ-5D-Y-5L followed that of Janssen et al. [32] for comparison of the EQ-5D-3L and EQ-5D-5L. Frequency of dimensions responses was summarised across age groups and health condition. Feasibility was examined by comparing the number of missing responses for the two EQ-5D-Y versions across age groups and health condition. Missing responses ≥ 5% per dimension was considered problematic since higher values may imply that the item is either not understood or does not make sense [35].

The ceiling and floor effects of the EQ-5D-Y-3L and EQ-5D-Y-5L were defined as the proportion of children/adolescents scoring “no problem” (11111) or the “most severe problems” (33333/55555) across all five dimensions, respectively. A reduction (absolute or relative) in ceiling or floor effect would suggest enhanced classification efficiency. The absolute reduction was calculated as the difference in proportion scoring 11111 or 33333/55555 from the EQ-5D-Y-3L to the EQ-5D-Y-5L. The relative reduction was calculated as ([ceiling/floor_EQ-5D-Y-3L_- ceiling/floor_EQ_5D-Y-5L_)]/ceiling/floor_EQ-5D-Y-3L_. It was hypothesized that the ceiling effect would be reduced both by age group and health condition when moving from the EQ-5D-Y-3L to the EQ-5D-Y-5L.

#### Redistribution properties of the EQ-5D-Y-3L to the EQ-5D-Y-5L

Paired dimension responses on the EQ-5D-Y-3L and EQ-5D-Y-5L were assessed for inconsistency across age groups and health condition using previously established criteria [16, 34]. A response pair was considered inconsistent if the EQ-5D-Y-5L response was more than two levels away from that of the EQ-5D-Y-3L. For example, a respondent choosing level 2 (some problems) in the EQ-5D-Y-3L but answering 5 (extreme problems) in the EQ-5D-Y-5L was considered inconsistent. The Chichewa versions are semantically equivalent to the English EQ-5D-Y versions such that level 3 on the EQ-5D-Y-3L (mavuto aakulu) matches level 4 on the EQ-5D-Y-5L (mavuto aakulu).

#### Discriminatory power

Discriminator power was evaluated using the Shannon Index (H′) and the Shannon Evenness Index (J′) informativity (absolute and relative) [3, 36]. The Shannon index has shown evidence of assessing spread of information within dimensions. The Shannon indices are defined as:

$$H^{\prime} = \mathop \sum \limits_{i = 1}^{L} p_{i} \log_{2} p_{i} \,\,{\text{and}}\,\,J^{\prime} = \frac{H^{\prime}}{{H^{\prime}_{\max } }}$$

where H′ is the absolute amount of informativity, L is the number of dimensions levels and p_i_ is the proportion of observations in the ith level where the EQ-5D-Y-3L has three levels and the EQ-5D-Y-5L has five levels. A higher H’ index reflects that the descriptive system has captured more information; the maximum H′index is 1.58 and 2.32 on the EQ-5D-Y-3L and EQ-5D-Y-5L, respectively [3]. It was anticipated that the H′index would increase for the EQ-5D-Y-5L compared to the EQ-5D-Y-3L. The Shannon Evenness index (J’) reflects the spread of the responses across levels regardless of the number of levels included in the descriptive system [3]. It was hypothesized that the J’index would remain the same or marginally decrease (as its not dependent on response levels) for the EQ-5D-Y-5L compared to the EQ-5D-Y-3L.

### Convergent validity

Convergent validity is the extent to which similar dimensions of two or more instruments are related. It is expected that similar dimensions will have a moderate to strong correlation. It was therefore hypothesized that the EQ-5D-Y-3L and EQ-5D-Y-5L sum and utility scores would be correlated (Pearson) with PedsQL™ 4.0 GCS total scale scores. It was further hypothesized that for both of the EQ-5D-Y versions, the dimensions of “mobility”, “doing usual activities”, and “feeling worried, sad or unhappy” would be correlated with PedsQL™ 4.0 GCS physical, school, and emotional functioning scores, respectively. It was hypothesized that the PedsQL™ 4.0 GCS correlation would be negative with the EQ-5D-Y levels sum score (better = lower score) but positive for the EQ-5D-Y utility score (better = higher score). A correlation ≥ 0.4 is considered moderate to strong [37].

### Discriminant validity

Discriminant validity is the extent to which unrelated dimensions between scales should not be similar. Further, it was anticipated that age, school grade and gender would not be factors in self-completion of the EQ-5D-Y-3L and EQ-5D-Y-5L. A Pearson correlation < 0.2 indicates lack of correlation. It was anticipated that there would be a lack of correlation between EQ-5D-Y-3L, EQ-5D-Y-5L sum and utility scores with age. It was also hypothesised that the correlation direction for sum score and age would be negative, and positive between age and utility scores. This is because a lower value is better for sum scores and vice versa for utility scores. No association at the 5% significance level was hypothesized between both the EQ-5D-Y-3L and EQ-5D-Y-5L sum and utility scores, with gender (t-test) and grade (one-way ANOVA). School grade was dichotomised based on general distribution and in line with the former scaling for primary school education in Malawi: grades 1–5 made group 1, grades 6–8 made group 2, and secondary/high school made group 3.

### Known-group validity

Known-group validity is the extent to which scores differ for two or more groups that are known to be different in some other aspects e.g., health status. It was hypothesised that for the two EQ-5D-Y versions, sum and utility scores would be worse for the sick compared to the healthy children. A t-test evaluated the relationship and the effect size was interpreted according to Cohen’s criterion: < 0.2 poor, 0.3–0.49 small, 0.5–0.8 moderate, and > 0.8 large [35, 38].

### Utility score performance (empirical validity)

The EQ-5D-Y-3L and EQ-5D-Y-5L are preference-based instruments used not only for measuring HRQoL but also in economic evaluation. As such, the EQ-5D measures the preference (value or utility) placed on specific health states [39]. It is important to evaluate how and to what extent the utilities generated by these instruments reflect revealed preferences, stated preferences or hypothesised preferences. In the absence of revealed preference and stated preference data, it was hypothesised that utility scores for both EQ-5D-Y versions would detect differences in external indicators of health status with the EQ-5D-Y-5L being more efficient at detecting differences (reflecting greater empirical validity) than the EQ-5D-Y-3L. It was further hypothesized that people would ‘prefer’ lower mild health problems.

The relative ability to assess external indicators of health status was investigated by comparing the utility scores with self-reported general health and the PedsQL™ 4.0 GCS total scale scores using the relative efficiency (RE) statistic. RE was defined as ‘the ratio of the square of the t-statistic of the comparator instrument over the square of the t-statistic of the reference instrument’ [40]. The EQ-5D-Y-5L acted as the comparator instrument and the EQ-5D-Y-3L as the referent since the latter has been widely used and psychometrically validated [7]. RE = 1.0 indicates that the EQ-5D-Y-5L has the same efficiency as the EQ-5D-Y-3L at detecting differences in health status; > 1.0 indicates that the EQ-5D-Y-5L is more efficient than the EQ-5D-Y-3L; and the converse is true [40].

Self-reported general health status was dichotomised using a frequency distribution [40] into two categories: (i) excellent or very good versus good or fair or poor, and (ii) excellent versus very good or good or fair or poor. The mean for the total scale scores provided a cut-off for the PedsQL™ 4.0 GCS such that less than mean, and mean and above formed two categories. The cut-off points used to create these dichotomous variables were necessarily arbitrary and may lead to different conclusions depending on which cut-offs are chosen. Therefore, in a series of sensitivity analyses, we dichotomised the self-reported general health status and PedsQL™ 4.0 GCS variables in alternative ways and replicated the analyses.

All empirical validity analyses were based on participants who completed both the EQ-5D-Y-5L and EQ-5D-Y-3L, thus any respondents with missing responses for either measure were excluded from this analysis. However, for the PedsQL™ 4.0 GCS, a volume of missing values of < 50% are taken into account as per the scoring algorithm [32]. There is a possibility that utility scores below 0 (which could lead to under predicting poorest heath states) would be different for the EQ-5D-Y-5L and EQ-5D-Y-3L since the utility scores are based on two different valuation models [29]. To overcome this,

## Results

### Participant characteristics

A total of 289 participants completed the EQ-5D-Y, EQ-5D-Y-5L, and PedsQL™ 4.0 GCS, aged 8–17 years (mean 13.6, median 14) as presented in Table 1. There were slightly more participants that were: females (56%), in primary school (60%) or ill (67%). The majority of the participants were adolescents (66%), and as expected all these were in high school.

**Table 1:** Participant characteristics

Characteristic	Sub-category	N (%)	Children (8–12 years) n (%)	Adolescents (13–17 years) n (%)
Participants	All	289	98 (34%)	191 (66%)
Gender*	Male	121 (44%)	39 (32%)	82 (68%)
	Female	153 (56%)	51 (33%)	102 (67%)
Health condition	healthy	95 (33%)	12 (13%)	83 (87%)
	acute	155 (54%)	85 (55%)	70 (45%)
	chronic	39 (13%)	1 (3%)	38 (97%)
School grade^#^	1–5	71 (25%)	53 (75%)	18 (25%)
	6–8	97 (35%)	40 (41%)	57 (59%)
	9–12	111 (40%)	0	111 (100%)

*Missing data: 15 (age group 1 = 8, age group 2 = 7)

^#^Missing data: 10 (5 in each age groups)

Age group 1 completed EQ-5D-Y, EQ-5D-Y-5L and PedsQL 4.0 child self-report

Age group 2 completed EQ-5D-Y, EQ-5D-Y-5L and PedsQL 4.0 teen self-report

### General instrument performance and feasibility

The EQ-5D-Y-3L had missing responses in all dimensions among children compared to none among adolescents (Table 2). For the EQ-5D-Y-5L, missing responses were observed in three dimensions among both children and adolescents. Across all respondents (aged 8–17 years), there were fewer dimensions with missing responses for the EQ-5D-Y-3L (two) compared to the EQ-5D-Y-5L (four).

**Table 2:** Proportion of reported problems in the EQ-5D-Y-3L and the EQ-5D-Y-5L

Dimension	EQ-5D-Y-5L			EQ-5D-Y-3L		
	8–12 years n (%)	13–17 years n (%)	8–17 years n (%)	8–12 years n (%)	13–17 years n (%)	8–17 years n (%)
*Mobility*						
No	67 (68%)	154 (81%)	221 (76%)	63 (64%)	164 (86%)	227 (79%)
A little bit	19 (19%)	21 (11%)	40 (14%)			
Some	5 (5%)	6 (3%)	11 (4%)	27 (28%)	21 (11%)	48(17%)
A lot	1(1%)	2 (1%)	3 (1%)	2 (2%)	2 (1%)	4 (1%)
Cannot	2 (2%)	0 (0%)	2 (1%)			
Missing	4 (4%)	8 (4%)	12 (4%)	6 (**6%**)	4 (2%)	10 (3%)
*Looking after myself*						
No	68 (68%)	164 (86%)	232 (80%)	68 (69%)	168 (88%)	236 (82%)
A little bit	18 (18%)	9 (5%)	27 (9%)			
Some	4 (4%)	7 (4%)	11 (4%)	18 (18%)	16 (8%)	34 (12%)
A lot	2 (2%)	0 (0%)	2 (1%)	3 (3%)	1 (1%)	4 (1%)
Cannot	2 (2%)	2 (1%)	4 (1%)			
Missing	4 (4%)	9 (**5%**)	13 (**5%**)	9 (**9%**)	6 (3%)	15 (**5%**)
*Usual activities*						
No	67 (68%)	150 (79%)	217 (75%)	64 (65%)	156 (82%)	220 (76%)
A little bit	16 (16%)	16 (8%)	32 (11%)			
Some	6 (6%)	14 (7%)	20 (7%)	22 (22%)	29 (15%)	51 (18%)
A lot	3 (3%)	2 (1%)	5 (2%)	4 (4%)	2 (1%)	6 (2%)
Cannot	1 (1%)	1 (1%)	2 (1%)			
Missing	5 (**5%**)	8 (4%)	13 (**5%**)	8 (**8%**)	4 (2%)	12 (4%)
*Pain or discomfort*						
No	55 (56%)	123 (64%)	178 (62%)	50 (51%)	137 (72%)	187 (65%)
A little bit	18 (18%)	36 (19%)	54 (19%)			
Some	15 (15%)	19 (10%)	34 (12%)	36 (37%)	46 (24%)	82 (28%)
A lot	3 (3%)	2 (1%)	5 (2%)	5 (5%)	4 (2%)	9 (3%)
Extreme	2 (2%)	1 (1%)	1 (0%)			
Missing	5 (**5%**)	10 (**5%**)	15 (**5%**)	7 (**7%**)	4 (2%)	11 (4%)
*Worried, sad or unhappy*						
No	62 (63%)	137 (72%)	199 (69%)	60 (61%)	142 (74%)	202 (70%)
A little bit	13 (13%)	30 (16%)	43 (15%)			
Some/quite	10 (10%)	12 (6%)	22 (7%)	24 (25%)	41 (22%)	65 (23%)
Really	3 (3%)	0 (0%)	3 (1%)	6 (6%)	1 (1%)	7 (2%)
Extremely	5 (5%)	3 (2%)	8 (3%)			
Missing	5 (**5%**)	9 (**5%**)	14 (5%)	8 (**8%**)	7 (4%)	15 (5%)
*EQ-VAS*						
Mean (SD)	82.5 (20.4)	89.2 (16.3)	87.0 (18.0)	82.7 (19.7)	89.2 (15.5)	87.0 (17.2)
Missing	5 (**5%**)	11 (**6%**)	23 (**8%**)	15 (**15%**)	13 (**7%**)	22 (**8%**)
Scores						
LSS Mean (SD)	7.7 (3.6)	6.5 (2.5)	6.9 (3.0)	6.8 (2.0)	5.9 (1.3)	6.2 (1.6)
US Mean (SD)	0.81 (0.28)	0.91 (0.17)	0.87 (0.22)	0.87 (0.14)	0.94 (0.10)	0.92 (0.11)

Bold indicates proportion of missing responses ≥ 5%

For the analysis based on health condition (Additional file 1: Table S1), both the EQ-5D-Y-3L and the EQ-5D-Y-5L had missing responses in all five dimensions among the acute (highest proportion) and chronically ill, but not in the healthy population.

The dimensions “looking after myself” and “having pain or discomfort” had the highest and lowest proportion of responses for both the EQ-5D-Y-3L and EQ-5D-Y-5L, respectively. This was similarly the case when the data were stratified by age and health condition. The dimensions of “mobility” (86%), “looking after myself” (88%), and “doing usual activities” (82%) had consistently higher proportions of “no problems” among adolescents, compared to children for the EQ-5D-Y-3L. Similarly, this was evident for the EQ-5D-Y-5L “mobility” (81%) and “looking after myself” (86%) dimensions.

The ceiling effect (11111) for all dimensions was generally reduced (9%) from the EQ-5D-Y-3L to EQ-5D-Y-5L for all participants (8–17 years) and among adolescents (Table 3). The greatest reduction in ceiling effect was in the ‘having pain or discomfort’ dimension for all participants (5%) and adolescents (11%). Among children, however, ceiling effects increased overall (48%) and for “having pain or discomfort” (10%). Overall, the floor effect (33333/55555) was mostly low except in the “having pain or discomfort” dimension (50–100%).

**Table 3:** Ceiling effect for the EQ-5D-Y-3L and EQ-5D-Y-5L across age groups and health condition

EQ-5D-Y-3L compared to EQ-5D-Y-5L	Children (n = 98)						Adolescents (n = 191)						All (N = 298)					
	Y-3L		Y-5L		AR	RR	Y-3L		Y-5L		AR	RR	Y-3L		Y-5L		AR	RR
	n	%	n	%			n	%	n	%			n	%	n	%		
*Ceiling effect (11111)*	25	27	36	40	− 13	− 48	104	55	96	50	5	9	128	44	132	46	− 2	− 5
Mobility (walking about)	67	68	67	68	0	0	164	86	154	81	5	6	227	79	221	76	3	4
Looking after myself	68	69	68	68	1	1	168	88	164	86	2	2	235	82	232	80	2	2
Doing usual activities	64	65	67	68	− 3	− 5	156	82	150	79	3	4	230	76	217	75	1	1
Having pain or discomfort	50	51	55	56	− 5	− 10	137	72	123	64	8	11	187	65	178	62	3	5
Feeling worried, sad, or unhappy	60	61	62	63	− 2	− 3	142	74	137	72	2	3	202	70	199	69	1	1
*Floor effect (11111)*	1	1	0	0	1	100	0	0	0	0	0	0	1	0	0	0	0	0
Mobility (walking about)	2	2	*2*	*2*	0	0	2	1	0	0	1	100	4	1	2	1	0	0
Looking after myself	3	3	*2*	*2*	1	33	1	1	2	1	0	0	4	1	4	1	0	0
Doing usual activities	4	4	*1*	*1*	3	75	2	1	1	1	0	0	6	2	2	1	1	50
Having pain or discomfort	5	5	*2*	*2*	3	60	4	2	1	1	1	50	9	3	1	0	3	100
Feeling worried, sad, or unhappy	6	6	*5*	*5*	1	17	7	4	9	5	− 1	− 25	15	5	14	5	0	0

EQ-5D-Y-3L compared to EQ-5D-Y-5L	Acute (n = 155)						Chronic (n = 39)						General population (n = 95)					
	Y-3L		Y-5L		AR	RR	Y-3L		Y-5L		AR	RR	Y-3L		Y-5L		AR	RR
	n	%	n	%			n	%	n	%			n	%	n	%		
*Ceiling effect (11111)*	41	26	44	28	− 2	− 8	29	74	33	85	− 11	− 15	58	61	58	61	0	0
Mobility (walking about)	103	67	98	63	4	6	35	90	33	85	5	6	89	94	90	95	− 1	− 1
Looking after myself	109	70	108	70	0	0	35	90	34	87	3	3	92	97	90	95	2	2
Doing usual activities	104	67	102	66	1	1	34	87	32	82	5	6	82	86	83	87	− 1	− 1
Having pain or discomfort	75	48	70	45	3	6	35	90	33	85	5	6	77	81	75	79	2	2
Feeling worried, sad, or unhappy	92	59	93	60	− 1	− 2	32	82	31	80	2	2	78	82	75	79	3	4
*Floor effect (11111)*	1	1	0	0	1	100	0	0	0	0	0	0	0	0	0	0	0	0
Mobility (walking about)	3	2	2	1	1	50	1	3	0	0	3	100	0	0	0	0	0	0
Looking after myself	4	3	4	3	0	0	0	0	0	0	0	0	0	0	0	0	0	0
Doing usual activities	6	4	2	1	3	75	0	0	0	0	0	0	0	0	0	0	0	0
Having pain or discomfort	8	5	3	2	3	60	1	3	0	0	3	100	0	0	0	0	0	0
Feeling worried, sad, or unhappy	7	5	7	5	0	0	0	0	0	0	0	0	0	0	1	1	− 1	0

AR, absolute reduction; RR, relative reduction

There was an increase in ceiling effect among the acute and chronically ill, but not among healthy participants. At a dimension level, the reduction was largest (6%) for “having pain or discomfort” in the acute and chronically ill. Additionally, there was a 6% ceiling effect reduction for “mobility” and “doing usual activities” among the chronically ill. Among the healthy participants, the largest ceiling effect reduction was in “feeling worried, sad or unhappy”. As with age, the floor effect, reporting most severe problems across all dimensions (33333/55555) ranged between 1 and 3% among the acutely and chronically ill. There was no floor effect reduction in any dimension for healthy participants.

#### Redistribution properties of the EQ-5D-Y-3L to the EQ-5D-Y-5L

Inconsistent responses were similar across dimensions and age groups (Additional file 2: Table S2) except for “looking after myself”, which had significantly higher inconsistency for 8–12 year olds (14%) compared to 13–18 year olds (3%). Across age groups and dimensions, the greatest inconsistency was in the “having pain or discomfort” dimension, 15% in children and 8% among adolescents. Similarly, for all respondents, the highest inconsistency (10%) was in the “having pain or discomfort” dimension. Across age groups and dimensions, this inconsistency happened mainly by moving from some problems on the EQ-5D-Y-3L to no problems on the EQ-5D-Y-5L.

#### Discriminatory power

Informativity of dimensions did not improve across all dimensions on the EQ-5D-Y-5L compared to the EQ-5D-Y-3L (Table 4). In contrast to what was hypothesized, the EQ-5D-Y-3L had a higher H’ index in all dimensions compared to the EQ-5D-Y-5L. It was anticipated that the J’ index (spread of responses) would remain the same or marginally decrease on the EQ-5D-Y-5L compared to the EQ-5D-Y-3L. The small difference (0.021–0.073) in the J’ index shows that the spread of responses on the EQ-5D-Y-5L and EQ-5D-Y-3L was distributed evenly. The EQ-5D-Y-3L had a higher J’ in all dimensions except “feeling worried, sad or unhappy” in comparison to the EQ-5D-Y-5L.

**Table 4:** Shannon Index (H′) and Shannon Evenness Index (J′) for the EQ-5D-Y-3L and EQ-5D-Y-5L dimensions

	EQ-5D-Y-5L		EQ-5D-Y-3L		Difference in H′	Difference in J′
	Shannon Index H′	Shannon Eveness Index J′	Shannon Index H′	Shannon Eveness Index J′		
Mobility	− 0.685	0.173	− 0.547	0.236	− 0.138	− 0.063
Looking after myself	− 0.616	0.184	− 0.476	0.257	− 0.140	− 0.073
Usual activities	− 0.748	0.171	− 0.594	0.235	− 0.154	− 0.064
Pain or discomfort	− 0.981	0.140	− 0.747	0.199	− 0.234	− 0.059
Worried, sad or unhappy	− 0.883	0.102	− 0.676	0.081	− 0.207	0.021
Average difference					− 0.175	− 0.048

### Convergent validity

Results of tests of convergent validity are summarised in Additional file 3: Table S3. Correlations were consistently in the right direction and met the criterion (≥ 0.4) for the EQ-5D-Y-3L and the teen PedsQL™ 4.0 GCS summary scores, and the EQ-5D-Y-5L with the child PedsQL™ 4.0 GCS summary scores. Most of the sub-scales also met the criterion of 0.4, except a few that did not (e.g., school/usual activities for all respondents (8–17-years), physical/mobility for child version and emotional/worried, sad or unhappy for the teen version).

### Discriminant validity

There was no significant difference (*p* > 0.05) between gender and EQ-5D-Y-3L nor EQ-5D-Y-5L sum scores or utility scores with exception of the direction of the relationship (Table 5).

**Table 5:** EQ-5D-Y-3L and EQ-5D-Y-5L discriminant validity by gender, school grade and age

Characteristic		Measure		Score*	Age 8–12 years				Age 13–17 years					Age 8–17 years				
					df	Mean diff	t	*p *value		df	Mean diff	t	*p *value		df	Mean diff	t	*p *value
Gender	EQ-5D-Y-3L		LSS		76	− 0.27	− 0.627	0.532		170	− 0.36	− 1.799	0.074		248	− 0.33	− 1.646	0.101
			US		76	0.02	0.678	0.500		170	0.03	1.871	0.063		248	0.02	1.707	0.089
	EQ-5D-Y-5L		LSS		86	0.39	0.558	0.578		174	− 0.05	− 0.143	0.886		262	0.09	0.252	0.802
			US		86	− 0.02	− 0.300	0.765		174	0.00	0.017	0.986		262	− 0.00	− 0.181	0.856
School grade^#^	EQ-5D-Y-3L		LSS		1	0.389	0.114	0.737		2	5.122	3.074	**0.049**		2	23.110	10.335	**< 0.001**
			US		1	0.000	0.013	0.911		2	0.030	3.848	**0.023**		2	0.123	11.450	**< 0.001**
	EQ-5D-Y-5L		LSS		1	1.539	0.146	0.703		2	28.585	4.741	**0.010**		2	56.435	7.491	**< 0.001**
			US		1	0.000	0.004	0.949		2	0.149	5.342	**0.006**		2	0.337	8.601	**< 0.001**

			Age 8–12 years				Age 13–17 years				Age 8–17 years			
Age	EQ-5D-Y-3L	LSS			0.1				− 0.2				**− 0.3**	
		US			− 0.1				0.2				**0.3**	
	EQ-5D-Y-5L	LSS			0.1				− 0.2				− 0.2	
		US			− 0.1				0.2				0.2	

*LSS, level sum score; US, utility score

^#^Grade group1 = grade 1–5; group2 = grade 6–8; group3 = grade 9–12; (age 8–12 years: grp1 = 53, grade grp 2 = 40; age 13–17 years: grp1 = 18, grade grp 2 = 57, grade grp3 = 111; age 8–17 years: grp1 = 71, grade grp 2 = 97, grade grp3 = 111). Bold indicates significance p < 0.05

There was a low Pearson correlation (0.1–0.2) and thus no association between age and both the sum and utility scores for the EQ-5D-Y-3L, and EQ-5D-Y-5L. The direction of correlation was as hypothesized in adolescents but not for children. However, this correlation between age and both the sum and utility scores improved (0.2–0.3) and was in the hypothesized direction in all respondents.

There was no evidence of difference between either EQ-5D-Y version’s sum (and utility) scores and school grade categories in children (*p* > 0.05), but this was statistically significant among adolescents (*p* < 0.05), and for all respondents (*p* < 0.001).

### Known-group validity

In children, although this might have skewed by a small number of healthy participants in this group (n = 12), the effect size was low (0.23) for the EQ-5D-Y-5L compared to high (− 1.15) for the EQ-5D-Y-3L. In adolescents, effect sizes were generally higher (> 0.5) suggesting reasonably good known-group validity (Additional file 4: Table S4). A similar effect size was observed for the utility scores between the healthy and sick groups although, as expected, the direction of the effect size was opposite to the sum scores.

### Empirical validity

Table 6 presents the relative efficiency statistics for the EQ-5D-Y-3L and EQ-5D-Y-5L over the dichotomous self-reported general health status and PedsQL™ 4.0 GCS measures, respectively. When the EQ-5D-Y-3L was referenced at 1.0, the EQ-5D-Y-5L was between 31 and 91% and between 5 and 44% less efficient than the EQ-5D-Y-3L at detecting differences in self-reported general health and the PedsQL™ 4.0 total scale score, respectively.

**Table 6:** Efficiency of the EQ-5D to detect differences in self-reported health status

Measure	Age	Categorisation of self-reported health status	Utility score^#^		t-test*		Relative efficiency
			mean	(SD)	t-statistic	*p *value	
EQ-5D-Y-3L	Age 8–12 years (n = 81)	Excellent or v. good	0.838	0.222	2.075	0.041	1.000
		Good or fair	0.746	0.175			
EQ-5D-Y 5L		Excellent or v. good	0.812	0.328	0.510	0.612	0.060
		Good or fair	0.780	0.243			
EQ-5D-Y-3L		Excellent	0.872	0.200	2.197	0.033	1.000
		v. good, good, fair or poor	0.766	0.205			
EQ-5D-Y 5L		Excellent	0.832	0.321	0.660	0.513	0.090
		v. good, good, fair or poor	0.783	0.284			
EQ-5D-Y-3L	Age 13–17 years (n = 172)	Excellent or v. good	0.903	0.137	0.148	0.883	1.000
		Good or fair	0.899	0.150			
EQ-5D-Y 5L		Excellent or v. good	0.911	0.175	0.123	0.902	0.693
		Good or fair	0.907	0.160			
EQ-5D-Y-3L		Excellent	0.933	0.116	2.205	0.029	1.000
		v. good, good, fair or poor	0.887	0.150			
EQ-5D-Y 5L		Excellent	0.924	0.210	0.704	0.483	0.102
		v. good, good, fair or poor	0.902	0.147			
EQ-5D-Y-3L	Combined ages 7–17 years (n = 253)	Excellent or v. good	0.883	0.169	1.733	0.085	1.000
		Good or fair	0.844	0.175			
EQ-5D-Y 5L		Excellent or v. good	0.881	0.235	0.702	0.484	0.164
		Good or fair	0.862	0.202			
EQ-5D-Y-3L		Excellent	0.913	0.150	3.027	0.003	1.000
		v. good, good, fair or poor	0.848	0.178			
EQ-5D-Y 5L		Excellent	0.895	0.253	0.945	0.346	0.098
		v. good, good, fair or poor	0.864	0.208			

Measure	Age	PedsQL 4.0 scale score	Mean	SD	t-statistic	*p *value	Relative efficiency
EQ-5D-Y-3L	Age 8–12 years (n = 81)	≥ 72.79	0.840	0.159	2.298	0.025	1.000
		< 72.79	0.727	0.249			
EQ-5D-Y 5L		≥ 72.79	0.865	0.223	2.237	0.030	0.948
		< 72.79	0.705	0.363			
EQ-5D-Y-3L	Age 13–17 years (n = 172)	≥ 78.68	0.946	0.087	3.837	0.000	1.000
		< 78.68	0.864	0.167			
EQ-5D-Y 5L		≥ 78.68	0.947	0.151	2.863	0.005	0.557
		< 78.68	0.872	0.177			
EQ-5D-Y-3L	Combined ages 7–17 years (n = 253)	≥ 76.81	0.918	0.114	4.716	0.000	1.000
		< 76.81	0.812	0.210			
EQ-5D-Y 5L		≥ 76.81	0.929	0.170	4.102	0.000	0.756
		< 76.81	0.808	0.265			

^#^US utilities

*Assuming equal variance

Restricting the analyses to participants with utility scores between 0 and 1 had the same outcome with the exception of the sensitivity analysis that dichotomised self-reported general health status as excellent versus very good, good or fair, which found that the EQ-5D-Y-5L was 736% more efficient than the EQ-5D-Y-3L at detecting differences in self-reported general health status. (Additional file 5: Table S5).

## Discussion

In this urban Malawian setting, both the EQ-5D-Y Chichewa versions demonstrated mixed evidence of instrument performance and feasibility, and validity. Both Chichewa versions demonstrated that they can be used with some limitations in missing responses, convergent and discriminant validity in this setting. The EQ-5D-Y-3L seems particularly suited for use in younger children (8–12 years) and the EQ-5D-Y-5L in adolescents (13–17 years). Other psychometric properties like test–retest reliability and responsiveness also need to be evaluated in this context.

Generally, the use of childhood preference-based HRQoL measures in sub-Saharan African settings is limited, as previously reported [41], and so the ability to generalize these findings in an African context is limited. Missing responses were relatively high in this study compared to other general population studies [9, 20]. The particularly high level of missing values among children (8–12 years) may point to sub-optimal reading skills in this age group in Malawi. This may indicate difficulty in providing good quality self-reported HRQoL assessment [24, 42] suggesting that younger children may benefit from an interviewer assisted approach [43].

The proportion reporting ‘no problems’ was similar between the EQ-5D-Y-3L and the EQ-5D-Y-5L, with the highest proportion for “looking after myself” and lowest in “having pain or discomfort” for both versions. This is consistent with findings from other studies with general population samples [9, 20, 42]. The proportion of ‘no problems’ was similarly spread across health conditions indicating that participants in this study may have had ‘milder’ health conditions. Like the adult EQ-5D-5L [44–46], the EQ-5D-Y-5L edged the EQ-5D-Y-3L in reducing ceiling effects, which may point to its improved sensitivity. However, the reduction but not elimination of the ceiling effect may indicate that this problem could be due to a true phenomenon as opposed to EQ-5D-Y-3L deficiency [18]. Further, the lack of ceiling effect reduction among the healthy group [18] is expected as this group should be experiencing fewer problems and may indicate that it is not necessary to include them in between-instruments ceiling effect comparisons in future studies.

The greatest proportion of inconsistencies was in the “having pain or discomfort” and “feeling worried, sad or unhappy” dimensions across age groups. As observed elsewhere [3, 20], these dimensions pertain to psychosocial concepts as opposed to physical aspect conveyed by the “mobility”, “looking after myself”, and “doing usual activities”. However, this variability originated from high ceiling effects, which may explain that among healthy participants (where reporting of no problems is expected) both versions work consistently well.

The discriminative power of the EQ-5D-Y-3L was marginally higher than that of the EQ-5D-Y-5L. This may imply that the informativity of dimensions does not improve on the EQ-5D-Y-5L in this setting. This has been observed in a previous study of idiopathic scoliosis [15], but is different from the general population [20] and those with other health conditions [47]. Considering that the application of Shannon indices is relatively new in HRQoL measurements, this might require further investigation.

The evidence for convergent validity shows that pre-specified criteria were met at scale but not at dimension level. This might imply that the EQ-5D-Y-3L and EQ-5D-Y-5L are best suited to assess physical functioning as opposed to other aspects of HRQoL. While the adult EQ-5D-5L has been found to be highly correlated with other health measures compared to the EQ-5D-3L [48–50], this was not the case with the two youth versions. These correlations were low to moderate, which is similar with other findings [12, 18, 46].

The discriminant ability of the EQ-5D-Y-3L and EQ-5D-Y-5L as regards gender and age is consistent with the adult EQ-5D-3L and EQ-5D-5L versions [45, 51]. The criterion was met for age groups but not across all respondents. Also, there were mixed relationships between sum and utility scores with age, which could not be established in this study but needs further research. While age has been associated with different scores for the EQ-5D-3L and EQ-5D-5L [45], this study did not find such differences between the EQ-5D-Y-3L and EQ-5D-Y-5L. Also, discriminant validity between both the EQ-5D-Y versions and school grade was met in children, but not among adolescents and across all respondents. This may indicate that years of education contributes to better completion and comprehension of questionnaires. Both the EQ-5D-Y-3L and EQ-5D-Y-5L showed evidence of known-group validity, which has been observed elsewhere [9, 17, 19, 21]. While the EQ-5D-Y-3L had the largest effect size in children, this was the case for the EQ-5D-Y-5L among adolescents. This study shows that the EQ-5D-Y-5L may be best suited for adolescents due to their ability to better distinguish responses, which is consistent with adult findings [52].

Tests of empirical validity demonstrated that the EQ-5D-Y-3L was generally more efficient than the EQ-5D-Y-5L at detecting hypothesised differences in external health status. This was surprising as the adult EQ-5D-5L has demonstrated greater relative efficiency compared to the EQ-5D-3L [53–55]. Our results may partly be due to the fact that the US EQ-5D-3L value set has additional interaction terms that may add more disutility to the weights compared to the US EQ-5D-5L value set. Also, the adult EQ-5D-5L has been found to overestimate health problems, leading to underestimation of utilities [4], which may have been the case with the sample in this study. Full understanding of why the EQ-5D-Y-3L outperformed the EQ-5D-Y-5L could benefit from future research.

Finally, it should be noted that there were no major differences in the psychometric tests focussed on utility values and the sum scores. The only difference was in the direction of the correlation. While the higher values were associated with better health outcomes for the utilities and vice versa for lower values, the opposite was true for the sum scores.

Limitations of this study include COVID-19 restrictions that led to collection of data in one wave and therefore test–retest reliability and responsiveness could not be evaluated. Secondly, preference-based value sets are not available for the EQ-5D-Y-5L and these have only recently been developed in three countries (at the time of doing this research) for the EQ-5D-Y-3L [56–58]. The use of adult values for childhood health states has been extensively discussed elsewhere [59]. The development of country-specific preference-based values for the EQ-5D-Y-5L is clearly an area that will benefit from further research although this may still be a limitation for the empirical validity i.e., whether EQ-5D reflect patient preferences in comparison to stated or revealed preferences.

## Conclusion

The two EQ-5D-Y versions established convergent and known-group validity among children and adolescents. Both versions had issues with missing values in younger children and discriminant validity by school grade as well as utilization of response options suggesting that the instruments can be used with caveats in this setting. These issues are likely not to be specific to Malawi as shown by evidence from elsewhere. Although the EQ-5D-Y-3L could be used across the age groups studied, it seems particularly suited (due to less nuanced responses) for use in younger children (8–12 years) whilst the EQ-5D-Y-5L seems particularly suited for use in adolescents (13–17 years) in Malawian contexts. Further psychometric testing for test re-test reliability and responsiveness is required, which could not be carried out in this study.

## Supplementary Information

**Additional file 1: Table S1.** Proportion of reported problems in the EQ-5D-Y-3L and the EQ-5D-Y-5L by health condition

**Additional file 2: Table S2.** Redistribution of the EQ-5D-Y-3L and EQ-5D-Y-5L dimension scores

**Additional file 3: Table S3.** Convergent validity of the EQ-5D-Y and EQ-5D-Y-5L with PedsQL™ 4.0 self-report sub-scale.

**Additional file 4: Table S4.** EQ-5D-Y-3L and EQ-5D-Y-5L sum score known group validity

**Additional file 5: Table S5.** Efficiency of the EQ-5D to detect differences in self-reported health status (utility set to between 0 and 1 only for both EQ-5D-Y and EQ-5D-Y-5L)

## Data Availability

The datasets generated and analysed during the current study are not publicly available as consent was not sought from participants for such but are available from the corresponding author on reasonable request.
