# Supplementary material for: Psychometric performance of the Chichewa versions of the EQ-5D-Y-3L and EQ-5D-Y-5L among healthy and sick children and adolescents in Malawi
Source: J Patient Rep Outcomes. 2023 Mar 9;7:22. doi: 10.1186/s41687-023-00560-4 (PMC9996597; doi:10.1186/s41687-023-00560-4)
Supplement: Supplementary file 1 — Additional file 1: Table S1. Proportion of reported problems in the EQ-5D-Y-3L and the EQ-5D-Y-5L by health condition [file 41687_2023_560_MOESM1_ESM.docx]

Supplementary Table 1 Proportion of reported problems in the EQ-5D-Y-3L and the EQ-5D-Y-5L by health condition

|  | | EQ-5D-Y-5L | | | | | | | EQ-5D-Y-3L | | | | | |
| --- | --- | --- | --- | --- | --- | --- | --- | --- | --- | --- | --- | --- | --- | --- |
|  | | Acute (n=155) | | | Chronic (n=39) | | Healthy (n=95) | | Acute (n=155) | | Chronic (n=39) | | Healthy (n=95) | |
|  |  | (n) | (%) | (n) | | (%) | (n) | (%) | (n) | (%) | (n) | (%) | (n) | (%) |
| **Mobility** |  |  |  |  | |  |  |  |  |  |  |  |  |  |
|  | no | 98 | 63% | 33 | | 85% | 90 | 95% | 103 | 67% | 35 | 90% | 89 | 94% |
|  | a little bit | 35 | 23% | 2 | | 5% | 3 | 3% |  |  |  |  |  |  |
|  | some | 8 | 5% | 2 | | 5% | 1 | 1% | 41 | 27% | 1 | 3% | 6 | 6% |
|  | a lot | 3 | 2% |  | |  |  |  | 3 | 2% | 1 | 3% |  |  |
|  | cannot | 2 | 1% |  | |  |  |  |  |  |  |  |  |  |
|  | missing | 9 | 6% | 2 | | 5% | 1 | 1% | 8 | 5% | 2 | 5% |  |  |
| **Looking after myself** | | | | | | | | | | | | | | |
|  | no | 108 | 70% | 34 | | 87% | 90 | 95% | 109 | 70% | 35 | 90% | 92 | 97% |
|  | a little bit | 25 | 16% |  | |  | 2 | 2% |  |  |  |  |  |  |
|  | some | 7 | 5% | 3 | | 8% | 1 | 1% | 29 | 19% | 2 | 5% | 3 | 3% |
|  | a lot | 1 | 1% |  | |  | 1 | 1% | 4 | 3% |  |  |  |  |
|  | cannot | 4 | 3% |  | |  |  |  |  |  |  |  |  |  |
|  | missing | 10 | 7% | 2 | | 5% | 1 | 1% | 13 | 8% | 2 | 5% |  |  |
| **Usual activities** | | | | | | | | | | | | | | |
|  | no | 102 | 66% | 32 | | 82% | 83 | 87% | 104 | 67% | 34 | 87% | 82 | 86% |
|  | a little bit | 26 | 17% | 2 | | 5% | 4 | 4% |  |  |  |  |  |  |
|  | some | 12 | 8% | 3 | | 8% | 5 | 5% | 35 | 23% | 4 | 10% | 12 | 13% |
|  | a lot | 3 | 2% |  | |  | 2 | 2% | 6 | 4% |  |  |  |  |
|  | cannot | 2 | 1% |  | |  |  |  |  |  |  |  |  |  |
|  | missing | 10 | 7% | 2 | | 5% | 1 | 1% | 10 | 7% | 1 | 3% | 1 | 1% |
| **Pain or Discomfort** | | | | | | | | | | | | | | |
|  | no | 70 | 45% | 33 | | 85% | 75 | 79% | 75 | 48% | 35 | 90% | 77 | 81% |
|  | a little bit | 39 | 25% | 1 | | 3% | 14 | 15% |  |  |  |  |  |  |
|  | some | 27 | 17% | 3 | | 8% | 4 | 4% | 62 | 40% | 2 | 5% | 18 | 19% |
|  | a lot | 4 | 3% |  | |  | 1 | 1% | 8 | 5% | 1 | 3% |  |  |
|  | extreme | 3 | 2% |  | |  |  |  |  |  |  |  |  |  |
|  | missing | 12 | 8% | 2 | | 5% | 1 | 1% | 10 | 7% | 1 | 3% |  |  |
| **Worried, Sad or Unhappy** | | | | | | | | | | | | | | |
|  | no | 93 | 60% | 31 | | 80% | 75 | 79% | 92 | 59% | 32 | 82% | 78 | 82% |
|  | a little bit | 28 | 18% | 3 | | 8% | 12 | 13% |  |  |  |  |  |  |
|  | some/quite | 13 | 8% | 3 | | 8% | 6 | 6% | 43 | 28% | 5 | 13% | 17 | 18% |
|  | really | 3 | 2% |  | |  |  |  | 7 | 5% |  |  |  |  |
|  | extremely | 7 | 5% |  | |  | 1 | 1% |  |  |  |  |  |  |
|  | Missing | 11 | 7% | 2 | | 5% | 1 | 1% | 13 | 8% | 2 | 5% |  |  |
| 11111 |  | 44 | 28% | 33 | | 85% | 58 | 61% | 41 | 26% | 29 | 74% | 58 | 61% |
| 33333/55555 | * | 0 |  | 0 | |  | 0 |  | 1 | 1% | 0 |  | 0 |  |
| EQ-VAS | mean (SD) | 83.44 (19.645) | | | 94.16 (10.756) | | 89.56 (16.553) | | 83.15 (19.246) | | 93.5 (8.655) | | 90.34 (15.106) | |
|  | missing | 16 |  | 2 | |  | 5 |  | 18 |  | 1 |  | 3 |  |

* health states with most severe problems across all dimensions (floor effect) for the EQ-5D-Y-3L and EQ-5D-Y-5L
