# Supplementary material for: Psychometric performance of the Chichewa versions of the EQ-5D-Y-3L and EQ-5D-Y-5L among healthy and sick children and adolescents in Malawi
Source: J Patient Rep Outcomes. 2023 Mar 9;7:22. doi: 10.1186/s41687-023-00560-4 (PMC9996597; doi:10.1186/s41687-023-00560-4)
Supplement: Supplementary file 2 — Additional file 2: Table S2. Redistribution of the EQ-5D-Y-3L and EQ-5D-Y-5L dimension scores [file 41687_2023_560_MOESM2_ESM.docx]

Supplementary Table 2 Redistribution of the EQ-5D-Y-3L and EQ-5D-Y-5L dimension scores

|  | Age 8-12 years | | | | | | | | Age 13-17 years | | | | | | | | Age 8-17 years | | | | | | |  |  |
| --- | --- | --- | --- | --- | --- | --- | --- | --- | --- | --- | --- | --- | --- | --- | --- | --- | --- | --- | --- | --- | --- | --- | --- | --- | --- |
| EQ-5D-Y-3L | EQ-5D-Y-5L | | | | | Total inconsistent responses n (%) | | EQ-5D-Y-5L | | | | | Total inconsistent responses n (%) | | | | EQ-5D-Y-5L | | | | Total inconsistent responses n(%) | |  |  |  |
| **Mobility** | no | a little bit | some | a lot | cannot | |  | | no | a little bit | some | a lot | | cannot |  | no | a little bit | some | a lot | cannot | |  | | | |
| No | 54 | 5 | ***2*** | ***1*** | ***0*** | | 14 (14%) | | 151 | 6 | ***1*** | ***0*** | | ***0*** | 3 (3%) | 205 | 11 | ***3*** | ***1*** | ***0*** | | 16 (6%) | | | |
| Some | ***9*** | 13 | 3 | 0 | ***1*** | |  |  | ***2*** | 13 | 4 | 1 | | ***0*** |  | ***10*** | 26 | 7 | 1 | ***1*** | |  |  |  |  |
| A lot | ***0*** | ***1*** | 0 | 0 | 1 | |  |  | ***0*** | ***0*** | 1 | 1 | | 0 |  | ***0*** | ***1*** | 1 | 1 | 0 | |  |  |  |  |
| **Looking after oneself** | | | | | | | | | | | | | | | | | | | | | | | | |  |
|  | no | a little bit | some | a lot | cannot | |  | | no | a little bit | some | a lot | | cannot |  | no | a little bit | some | a lot | cannot | |  | | | |
| No | 60 | 4 | ***2*** | ***1*** | ***0*** | | 5 (5%) | | 158 | 3 | ***2*** | ***0*** | | ***0*** | 7 (4%) | 218 | 7 | ***3*** | ***1*** | ***0*** | | 11 (4%) | | | |
| Some | ***2*** | 13 | 2 | 0 | ***0*** | |  |  | ***3*** | 6 | 4 | 0 | | ***2*** |  | ***5*** | 19 | 6 | 0 | ***2*** | |  |  |  |  |
| A lot | ***0*** | ***0*** | 0 | 1 | 2 | |  |  | ***0*** | ***0*** | 1 | 0 | | 0 |  | ***0*** | ***0*** | 1 | 1 | 1 | |  |  |  |  |
| **Usual activities** | | | | | | | | | | | | | | | | | | | | | | | | |  |
|  | no | a little bit | some | a lot of | cannot | |  | | no | a little bit | some | a lot of | | cannot |  | no | a little bit | some | a lot of | cannot | |  | | | |
| No | 56 | 5 | ***1*** | ***1*** | ***0*** | | 11 (11%) | | 142 | 5 | ***3*** | ***1*** | | ***1*** | 12 (6%) | 198 | 10 | ***4*** | ***2*** | ***1*** | | 23 (8%) | | | |
| Some | ***5*** | 9 | 5 | 1 | ***1*** | |  |  | ***7*** | 10 | 11 | 0 | | ***0*** |  | ***12*** | 19 | 16 | 0 | ***1*** | |  |  |  |  |
| A lot | ***1*** | ***2*** | 0 | 1 | 0 | |  |  | ***0*** | ***0*** | 0 | 1 | | 0 |  | ***1*** | ***2*** | 0 | 1 | 0 | |  |  |  |  |
| **Pain or Discomfort** | | | | | | | | | | | | | | | | | | | | | | | | |  |
|  | no | a little bit | some | a lot | extreme | |  | | no | a little bit | some | a lot | | extreme |  | no | a little bit | some | a lot | extreme | |  | | | |
| No | 43 | 2 | ***3*** | ***1*** | ***0*** | | 15 (15%) | | 116 | 8 | ***7*** | ***1*** | | ***0*** | 15 (8%) | 159 | 10 | ***10*** | ***2*** | ***0*** | | 30 (10%) | | | |
| Some | ***9*** | 14 | 10 | 2 | ***0*** | |  |  | ***7*** | 27 | 9 | 0 | | ***0*** |  | ***16*** | 40 | 19 | 2 | ***0*** | |  |  |  |  |
| A lot | ***1*** | ***1*** | 1 | 0 | 2 | |  |  | ***0*** | ***0*** | 2 | 1 | | 1 |  | ***1*** | ***1*** | 3 | 1 | 2 | |  |  |  |  |
| **Worried, Sad or Unhappy** | | | | | | | | | | | | | | | | | | | | | | | | |  |
|  | no | a little bit | quite | really | extreme | |  | | no | a little bit | quite | really | | extreme |  | no | a little bit | quite | really | extreme | |  | | | |
| No | 49 | 7 | ***1*** | ***1*** | ***1*** | | 13 (13%) | | 128 | 7 | ***0*** | ***2*** | | ***0*** | 12 (6%) | 177 | 14 | ***3*** | ***1*** | ***1*** | | 25 (9%) | | | |
| A bit | ***8*** | 5 | 8 | 1 | ***1*** | |  | | ***9*** | 21 | 0 | 9 | | ***1*** |  | ***17*** | 26 | 16 | 1 | ***2*** | |  | | | |
| Very | ***0*** | ***1*** | 1 | 1 | 3 | |  | | ***0*** | ***0*** | 0 | 0 | | 1 |  | ***0*** | ***1*** | 1 | 1 | 3 | |  | | | |
|  |  |  |  |  |  | |  | |  |  |  |  | |  |  |  |  |  |  |  | |  | | | |
