# Supplementary material for: Psychometric performance of the Chichewa versions of the EQ-5D-Y-3L and EQ-5D-Y-5L among healthy and sick children and adolescents in Malawi
Source: J Patient Rep Outcomes. 2023 Mar 9;7:22. doi: 10.1186/s41687-023-00560-4 (PMC9996597; doi:10.1186/s41687-023-00560-4)
Supplement: Supplementary file 3 — Additional file 3: Table S3. Convergent validity of the EQ-5D-Y and EQ-5D-Y-5L with PedsQL™ 4.0 self-report sub-scale. [file 41687_2023_560_MOESM3_ESM.docx]

Supplementary Table 3 Convergent validity of the EQ-5D-Y and EQ-5D-Y-5L with PedsQL^TM^ 4.0 self-report sub-scale.

| Measure |  | PedsQL^TM^ 4.0 child self-report | | | |  | PedsQL^TM^ 4.0 teen self-report | | | |
| --- | --- | --- | --- | --- | --- | --- | --- | --- | --- | --- |
|  | Sub-scale  Dimension | Emotional Functioning | School Functioning | Physical Health Summary | Total Scale score |  | Emotional Functioning | School Functioning | Physical Health Summary | Total Scale score |
| EQ-5D-Y-3L | Mobility |  |  | -0.3^*^ |  |  |  |  | **-0.4^**^** |  |
|  | Doing usual activities |  | -0.3 |  |  |  |  | -0.2 |  |  |
|  | Worried, sad or unhappy | **-0.4** |  |  |  |  | -0.3 |  |  |  |
|  | EQ-5D-Y sum score |  |  |  | **-0.4^**^** |  |  |  |  | **-0.4^**^** |
|  | Utility scores |  |  |  | **0.4^**^** |  |  |  |  | **0.4^**^** |
| EQ-5D-Y-5L | Mobility |  |  | -0.3^*^ |  |  |  |  | **-0.4** |  |
|  | Doing usual activities |  | -0.2 |  |  |  |  | -0.2 |  |  |
|  | Worried, sad or unhappy | -0.3 |  |  |  |  | **-0.4** |  |  |  |
|  | EQ-5D-Y-5L sum score |  |  |  | **-0.4^**^** |  |  |  |  | **-0.4^**^** |
|  | Utility scores |  |  |  | **0.4^**^** |  |  |  |  | **0.4^**^** |

**. Correlation is significant at the 0.01 level (2-tailed); *. Correlation is significant at the 0.05 level (2-tailed). Bold represents evidence of correlation ≥0.4 above the threshold
