# Supplementary material for: Psychometric performance of the Chichewa versions of the EQ-5D-Y-3L and EQ-5D-Y-5L among healthy and sick children and adolescents in Malawi
Source: J Patient Rep Outcomes. 2023 Mar 9;7:22. doi: 10.1186/s41687-023-00560-4 (PMC9996597; doi:10.1186/s41687-023-00560-4)
Supplement: Supplementary file 4 — Additional file 4: Table S4. EQ-5D-Y-3L and EQ-5D-Y-5L sum score known group validity [file 41687_2023_560_MOESM4_ESM.docx]

Supplementary Table 4 EQ-5D-Y-3L and EQ-5D-Y-5L sum score known group validity

|  |  | Age 8-12 years  (N=98, healthy=12, sick=86) | | | | | | | | Age 13-17 years  (N=191, healthy=83, sick =108) | | | | | | | |  | Age 8-17 years  (N=289, healthy=95, sick =194) | | | | |
| --- | --- | --- | --- | --- | --- | --- | --- | --- | --- | --- | --- | --- | --- | --- | --- | --- | --- | --- | --- | --- | --- | --- | --- |
|  |  | t-statistic ^#^ | | |  |  |  | | t-statistic ^#^ | | | |  | |  | |  |  | t-statistic ^#^ | |  |  |  |
| Measure |  | t | p-value | Mean Diff | | SD | Effect size ^¶^ | t | | | p-value | Mean Diff | | SD | | Effect size ^¶^ | |  | t | p-value | Mean Diff | SD | Effect size ^¶^ |
| EQ-5D-Y-3L | LSS | 1.184 | 0.240 | 0.951 | | 3.729 | **0.3** | -2.757 | | | 0.006 | -0.536 | | 0.884 | | **-0.6** | |  | -4.499 | <0.001 | -0.919 | 0.920 | **-1.0** |
|  | US | -0.702 | 0.455 | -0.038 | | 0.217 | **-0.2** | 2.699 | | | 0.008 | 0.036 | | 0.0613 | | **0.6** | |  | 4.587 | <0.001 | 0.064 | 0.064 | **1.0** |
|  |  |  |  |  | |  |  |  | | |  |  | |  | |  | |  |  |  |  |  |  |
| EQ-5D-Y-5L | LSS | 2.364 | 0.020 | 3.100 | | 6.256 | **0.5** | -3.480 | | | <0.001 | -1.262 | | 1.220 | | **-1.0** | |  | -3.972 | <0.001 | -1.470 | 1.873 | **-0.8** |
|  | US | -2.267 | 0.026 | -0.233 | | 0.520 | **-0.5** | -3.548 | | | <0.001 | 0.089 | | 0.073 | | **1.2** | |  | 3.825 | <0.001 | 0.1049 | 0.140 | **0.8** |
|  |  |  |  |  | |  |  |  | | |  |  | |  | |  | |  |  |  |  |  |  |

LSS: level sum score; US: utility score

*2 and 5 did not complete EQ-5D-Y and EQ-5D-Y-5L respectively

^#^ assuming equal variance

^¶^ effect size designated as <0.2 poor, 0.3-0.49 small, 0.5-0.8 moderate, and >0.8 large
