# Supplementary material for: Psychometric performance of the Chichewa versions of the EQ-5D-Y-3L and EQ-5D-Y-5L among healthy and sick children and adolescents in Malawi
Source: J Patient Rep Outcomes. 2023 Mar 9;7:22. doi: 10.1186/s41687-023-00560-4 (PMC9996597; doi:10.1186/s41687-023-00560-4)
Supplement: Supplementary file 5 — Additional file 5: Table S5. Efficiency of the EQ-5D to detect differences in self-reported health status (utility set to between 0 and 1 only for both EQ-5D-Y and EQ-5D-Y-5L) [file 41687_2023_560_MOESM5_ESM.docx]

Supplementary Table 5 Efficiency of the EQ-5D to detect differences in self-reported health status (utility set to between 0 and 1 only for both EQ-5D-Y and EQ-5D-Y-5L)

| MEASURE | AGE | Categorisation of self-reported health status and PedsQL 4.0 scale score | Utility score^#^ | |  | t-test* | | Relative efficiency |
| --- | --- | --- | --- | --- | --- | --- | --- | --- |
|  |  |  | mean | (SD) |  | t-statistic | p-value |  |
| EQ-5D-Y | age 8-12yrs (n=78) | excellent or v. good | 0.855 | 0.175 |  | 2.521 | 0.014 | 1.000 |
|  |  | good or fair | 0.755 | 0.171 |  |  |  |  |
| EQ-5D-Y-5L |  | excellent or v. good | 0.863 | 0.219 |  | 1.206 | 0.232 | 0.229 |
|  |  | good or fair | 0.805 | 0.200 |  |  |  |  |
| EQ-5D-Y |  | excellent | 0.867 | 0.202 |  | 1.688 | 0.099 | 1.000 |
|  |  | v. good, good, fair or poor | 0.789 | 0.164 |  |  |  |  |
| EQ-5D-Y 5L |  | excellent | 0.870 | 0.262 |  | 0.786 | 0.437 | 0.217 |
|  |  | v. good, good, fair or poor | 0.824 | 0.185 |  |  |  |  |
| EQ-5D-Y | age 13-17yrs (n=171) | excellent or v. good | 0.904 | 0.138 |  | 0.184 | 0.854 | 1.000 |
|  |  | good or fair | 0.899 | 0.150 |  |  |  |  |
| EQ-5D-Y-5L |  | excellent or v. good | 0.920 | 0.140 |  | 0.532 | 0.596 | 8.360 |
|  |  | good or fair | 0.907 | 0.160 |  |  |  |  |
| EQ-5D-Y |  | excellent | 0.935 | 0.116 |  | 2.306 | 0.023 | 1.000 |
|  |  | v. good, good, fair or poor | 0.887 | 0.150 |  |  |  |  |
| EQ-5D-Y-5L |  | excellent | 0.945 | 0.144 |  | 1.793 | 0.076 | 0.605 |
|  |  | v. good, good, fair or poor | 0.902 | 0.147 |  |  |  |  |
| EQ-5D-Y | combined ages 7-17yrs (n=249) | excellent or v. good | 0.890 | 0.151 |  | 1.900 | 0.059 | 1.000 |
|  |  | good or fair | 5.000 | 0.171 |  |  |  |  |
| EQ-5D-Y 5L |  | excellent or v. good | 0.904 | 0.169 |  | 1.393 | 0.165 | 0.538 |
|  |  | good or fair | 0.871 | 0.181 |  |  |  |  |
| EQ-5D-Y |  | excellent | 0.914 | 0.151 |  | 2.736 | 0.007 | 1.000 |
|  |  | v. good, good, fair or poor | 0.856 | 0.160 |  |  |  |  |
| EQ-5D-Y-5L |  | excellent | 0.921 | 0.191 |  | 1.744 | 0.084 | 0.406 |
|  |  | v. good, good, fair or poor | 0.878 | 0.163 |  |  |  |  |
| EQ-5D-Y | age 8-12yrs (n=78) | ≥ 73.10 | 0.866 | 0.129 |  | 3.255 | 0.002 | 1.000 |
|  |  | < 73.10 | 0.733 | 0.206 |  |  |  |  |
| EQ-5D-Y-5L |  | ≥ 73.10 | 0.908 | 0.148 |  | 3.103 | 0.003 | 0.909 |
|  |  | < 73.10 | 0.754 | 0.252 |  |  |  |  |
| EQ-5D-Y | age 13-17yrs (n=171) | ≥ 78.64 | 0.947 | 0.086 |  | 3.917 | 0.000 | 1.000 |
|  |  | < 78.64 | 0.864 | 0.167 |  |  |  |  |
| EQ-5D-Y-5L |  | ≥ 78.64 | 0.960 | 0.085 |  | 3.963 | 0.000 | 1.024 |
|  |  | < 78.64 | 0.872 | 0.177 |  |  |  |  |
| EQ-5D-Y | combined ages 7-17yrs (n=249) | ≥ 76.93 | 0.923 | 0.107 |  | 4.986 | 0.000 | 1.000 |
|  |  | < 76.93 | 0.819 | 0.191 |  |  |  |  |
| EQ-5D-Y-5L |  | ≥ 76.93 | 0.945 | 0.109 |  | 5.096 | 0.000 | 1.045 |
|  |  | < 76.93 | 0.829 | 0.212 |  |  |  |  |

^#^ US tariffs

*assuming equal variance
